# Changes in Cosmetics Use during Pregnancy and Risk Perception by Women

**DOI:** 10.3390/ijerph13040383

**Published:** 2016-03-30

**Authors:** Cécile Marie, Sophie Cabut, Françoise Vendittelli, Marie-Pierre Sauvant-Rochat

**Affiliations:** 1 EA 4681, PEPRADE (Périnatalité, Grossesse, Environnement, PRAtiques médicales et Developement), University of Auvergne, 28 Place Henri-Dunant BP 38, Clermont-Ferrand 63001, France; fvendittelli@chu-clermontferrand.fr (F.V.); m-pierre.sauvant-rochat@udamail.fr (M.-P.S.-R.); 2 Department of Public Health and Environment, Faculty of Pharmacy, University of Auvergne, 28 Place Henri-Dunant BP 38, Clermont-Ferrand 63001, France; sophie.cabut@laposte.net; 3 Department of Obstetrics and Gynecology, Clermont-Ferrand University Hospital Center, 58 Rue Montalembert, Clermont-Ferrand Cedex 1 63003, France; 4 AUDIPOG (Association des Utilisateurs de Dossiers informatisés en Pédiatrie, Obstétrique et Gynécologie), RTH Laennec Medical University, 7 Rue Guillaume Paradin, Lyon Cedex 08 69372, France

**Keywords:** cosmetics, personal care products, pregnancy, risk perception, health education, endocrine disruptors

## Abstract

Cosmetic products contain various chemical substances that may be potential carcinogen and endocrine disruptors. Women’s changes in cosmetics use during pregnancy and their risk perception of these products have not been extensively investigated. The main objective of this study was to describe the proportion of pregnant women changing cosmetics use and the proportion of non-pregnant women intending to do so if they became pregnant. The secondary objectives were to compare, among the pregnant women, the proportions of those using cosmetics before and during pregnancy, and to describe among pregnant and non-pregnant women, the risk perception of these products. A cross-sectional study was carried out in a gynaecology clinic and four community pharmacies. One hundred and twenty-eight women (60 non-pregnant and 68 pregnant women) replied to a self-administered questionnaire. Cosmetics use was identified for 28 products. The results showed that few women intended to change or had changed cosmetics use during pregnancy. Nail polish was used by fewer pregnant women compared to the period before pregnancy (*p* < 0.05). Fifty-five percent of the women considered cosmetics use as a risk during pregnancy and 65% would have appreciated advice about these products. Our findings indicate that all perinatal health professionals should be ready to advise women about the benefits and risks of using cosmetics during pregnancy.

## 1. Introduction

The European Union Cosmetics Directive defines a cosmetic product or personal care product (PCP) as “any substance or mixture intended to be placed in contact with the external parts of the human body (epidermis, hair system, nails, lips and external genital organs) or with the teeth and the mucous membranes of the oral cavity with a view exclusively or mainly to cleaning them, perfuming them, changing their appearance, protecting them, keeping them in good condition or correcting body odors” [1]. Cosmetic products are widely used in daily life. They contain various chemical substances such as phthalates and other plasticizers, bisphenol A, parabens, benzophenones (ultraviolet filters), polycyclic musks, triclosan (antimicrobials), dioxane, organic solvents, pigments, formaldehyde, and heavy metals that serve as active ingredients, solvents, preservatives and additives to improve their efficacy and increase the duration of their effect [2,3,4,5]. Some of these chemicals have a clear benefit. For example, ultraviolet filters protect human skin from direct exposure to deleterious ultraviolet radiation.

However, these molecules can enter humans via different routes of exposure. Most cosmetic products are directly applied on the skin and their ingredients can cross the cutaneous barrier to reach the systemic circulation. Exposure can also occur by contact with the mucous membranes, by ingestion (as with lipstick, for example) and by inhalation (for cosmetics in the form of aerosols, or during application of a varnish) [6,7]. Numerous authors have shown that the use of cosmetics is associated with increased levels of exposure to phenols including benzophenone-3 [8,9], parabens [8,10,11,12,13] and triclosan [13], and plasticizers, especially diethyl phthalate (DEP) [8,10,11,14,15,16,17] and triphenyl phosphate (TPHP) [18] in humans.

To our knowledge, there is no current evidence that these effects are directly linked to the use of cosmetics [3]. Nevertheless, the endocrine disruptive effects of some of these molecules have been shown in numerous experimental models [19,20,21,22,23,24,25,26,27] and in human studies [28,29,30,31,32,33].

Pregnant women are particularly vulnerable to the potential risks of the endocrine disruptors contained in cosmetics. First, cosmetics use is far more common among women than men [34,35]. Second, pregnancy is a vulnerable time for the development of the embryo and fetus because of their immature metabolism. Finally, exposure to endocrine disruptors can be changed during this period by changes in life habits such as a different diet and use of cosmetics. Some studies have been conducted in the USA and in Canada on the use of cosmetics during pregnancy [11,16,17,36,37] but none, however, dealt with changes in habits such as stopping, decreasing or increasing the use of a product because of pregnancy. Nor has there been any assessment of women’s risk perception of cosmetics use during pregnancy. Barrett *et al.* (2014) showed that pregnant women who believe that environmental chemicals are dangerous had healthy behaviors, especially food behaviors, and chose “eco-friendly” PCPs [38]. However, their study assessed the perception of chemical products “in general” (including products in food, for example) and not specifically those contained in PCPs.

To our knowledge, there exist no reports on what advice, if any, is given by health professionals to women about cosmetic use during pregnancy. Changes in use habits of cosmetic products during pregnancy, risk perception of these products, and support and advice from perinatal health professionals (obstetricians, midwives, general practitioners, community pharmacists) are essential to any prevention and awareness strategy aimed at limiting the exposure of pregnant women to endocrine disruptors. In France, cosmetic products are frequently sold in community pharmacies, and pharmacists can therefore play an important part in advising and informing pregnant women.

The main objective of this study was to describe the proportion of pregnant women who changed their use of cosmetic products, and the proportion of non-pregnant women who would expect to do so if they became pregnant. The secondary objectives were to compare, among the pregnant women, the proportions of those using cosmetics before and during their pregnancy, and to describe, among pregnant and non-pregnant women, the criteria for deciding to use cosmetics before and during pregnancy, the risk perception of the use of these products, and the provision of advice by health professionals.

## 2. Subjects and Method

### 2.1. Subjects

The study population was made up of pregnant women and young women of childbearing age. The study was conducted in four community pharmacies and a private gynaecology clinic located in two adjacent French departments (Loire and Haute-Loire). To be eligible for inclusion, the women had to be aged between 18 and 45 years (≥18 years and <45 years), have a good command of French and give informed consent to take part. One hundred and twenty-eight women were included (60 non-pregnant women (NPW) and 68 pregnant women (PW)).

In accordance with French human research law, this study was exempt from Institutional Review approval because our database included no nominative data and the survey was not an interventional research study.

### 2.2. Method

A cross-sectional study was performed between 31 March 2015 and 1 July 2015. The women were informed of the aim of the survey and what it would entail (completion of an anonymous self-administered questionnaire) by a health professional taking part in the study, either a community pharmacist or a gynaecologist.

Data were collected by a standardized, anonymous, self-administered questionnaire. They comprised the socio-demographic and obstetrical characteristics of the women enrolled, their use of cosmetics outside of pregnancy (products used and criteria of choice), changes in use following pregnancy (PW subgroup) or intended changes in the event of pregnancy (NPW subgroup), risk perception of the use of cosmetic products, and previous advice about these products given by a health professional. The suggested criteria of choice were: price, ingredients, odor, appearance, recommendation of a professional or friend, habit, and preference for a range or brand. Use habits were established for 28 cosmetics: nine PCPs for general hygiene (shower gel, solid soap, dermatological soap, intimate hygiene product, body scrub, body lotion or cream, deodorant (spray or non-spray), perfume and bronzers), five PCPs for face hygiene (facial cleanser, day face cream, night face cream, facial scrub and facial mask), three PCPs for hair hygiene (shampoo, hair mask and hair dye (professional and home hair dye without distinction)) and 11 make-up products (foundation, blush, mascara, eye-liner, eye pencil, eye shadow, lipstick, lip pencil, make-up remover, nail polish and nail polish remover). The use of cosmetics outside of pregnancy was assessed on the basis of the women’s general use habits (and not use during the 24 or 48 h before they completed the questionnaire) in order to characterize cosmetics such as nail polish and hair dye that are not necessarily used daily. For example, use of shower gel was assessed by the following question “Outside of pregnancy, do you regularly use *shower gel*?” (Yes/No). The same question was asked for the other 27 PCPs. For each product, women who spontaneously responded "Yes" were considered as using the product regularly, irrespective of the frequency of use. The changes in use during pregnancy (PW subgroup) or intended changes in the event of pregnancy (NPW subgroup) were assessed by the following question: “Since the beginning of pregnancy (or in the event of pregnancy), have you changed (or will you change) the use of…?” (Yes/No). This question was asked for the 28 PCPs. The questionnaire addressed to the PW group also concerned changes in cosmetics use during pregnancy, such as giving up the use of a product, decreasing the use, replacing a product by something considered less harmful, increasing use of a product and using a new one.

In the subgroup of PW, the proportions of women having used cosmetics before their pregnancy and the proportions of those using cosmetics during pregnancy were compared.

### 2.3. Statistical Analysis

The qualitative variables were compared in the PW and NPW subgroups with Pearson’s Chi-square or Fisher’s exact test, as appropriate. The quantitative variables were compared with Student’s *t*-test or a Mann–Whitney test, as appropriate.

A cross-over analysis using McNemar’s Chi-squared test (with continuity correction if appropriate) was performed in the PW subgroup to compare the proportions of cosmetics users before and after pregnancy.

Significance was defined as *p* < 0.05. Statistical analyses were performed with R statistical software, version 2.15.2 (R Development Core Team, Vienna, Austria, 2012).

## 3. Results

### 3.1. Women’s Characteristics

The mean age of the women in our sample was 30.5 ± 5.8 years and more than half of them had (51.2%) already had one pregnancy (Table 1). In both subgroups (NPW and PW), most women had attended university (74.8%) and were mainly working in the intermediate professions (35.9%) or as salaried employees (31.3%). Compared with the NPW, PW were more likely to live in a town of fewer than 5000 inhabitants (*p* < 0.01) and less likely to have received chronic treatment before pregnancy (*p* = 0.05) (Table 1). In the PW subgroup (*n* = 68), the mean gestational age of the pregnancy was 26.3 ± 8.2 weeks: 10.3% of the women were in the first trimester of pregnancy, 44.1% in the second and 45.6% in the third.

### 3.2. Proportion of Women Using Cosmetics Outside of Pregnancy

The proportions of women using cosmetics outside of pregnancy are given in Table 2 for PCPs and in Table 3 for make-up products. The proportions varied widely according to the type of cosmetic product. The use of dermatological soap was significantly greater among NPW (25.9%) than among PW (10.3%) (*p* = 0.03). The use of other cosmetics before pregnancy did not differ between the two subgroups.

### 3.3. Criteria of Choice of Cosmetics Outside of Pregnancy

**Figure 1:**
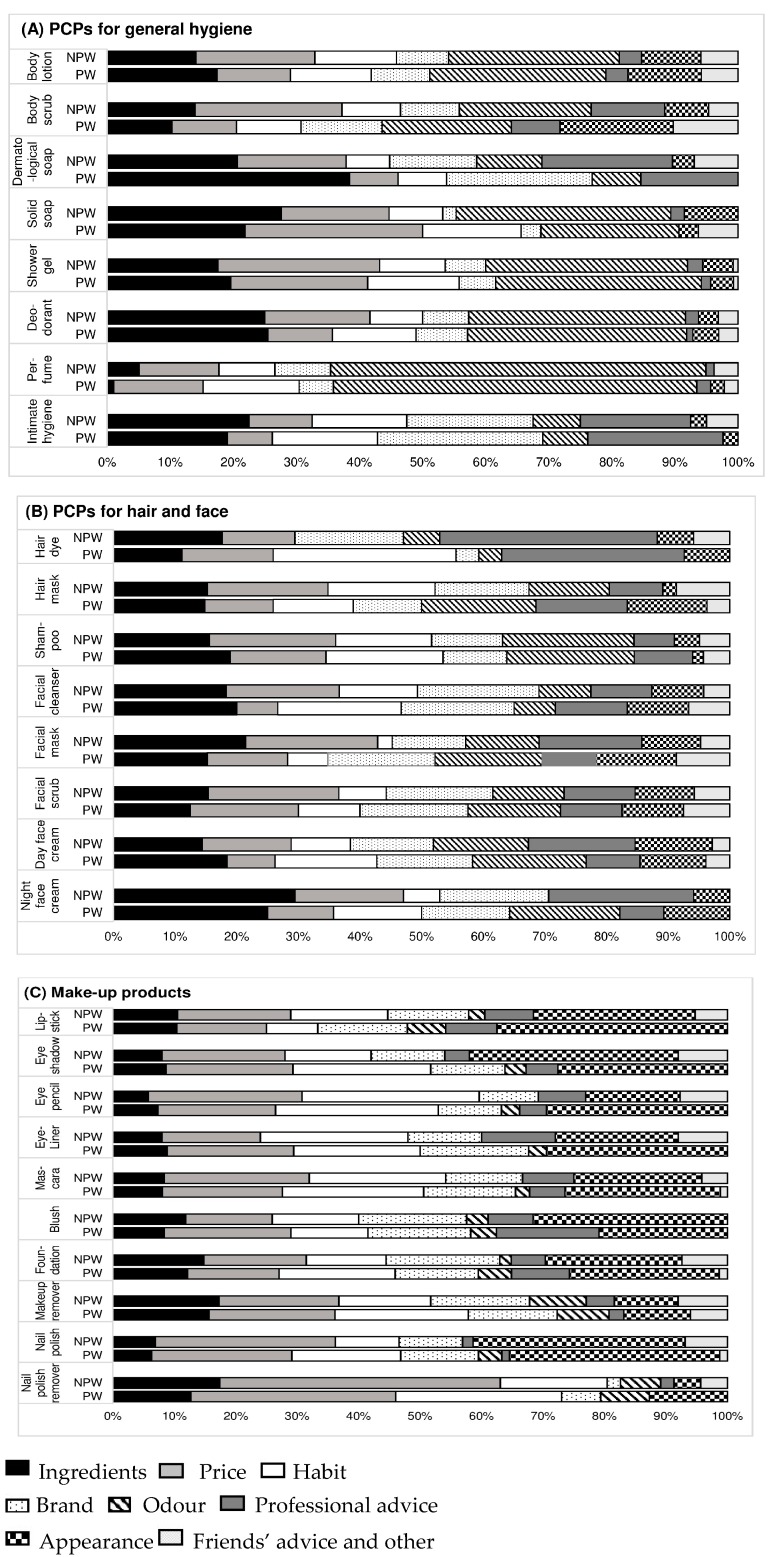
Criteria of choice of products outside of pregnancy. (**A**) PCPs for general hygiene; (**B**) PCPs for hair and face; (**C**) make-up products. Abbreviations: NPW, non-pregnant women; PCP: personal care product; PW: pregnant women.

For general hygiene PCPs, the first criterion of choice in both subgroups (NPW and PW) was odor (except for intimate hygiene products and dermatological soap), followed by price and ingredients (Figure 1). For hair and face PCPs, the criteria of choice varied overall, with a slight predominance of ingredients and price, apart from hair dyes, for which professional advice was the most common deciding factor (33% of the women). For make-up products, price, appearance and habit were the decisive criteria in both subgroups (Figure 1). Cosmetics for which the ingredients were most often cited as the criterion of choice were dermatological soap (71.4%), night facial cream (58.3%), shower gel (40.9%) and deodorant (40.3%). Cosmetics for which the ingredients were the least often cited as the criterion of choice (less than 10% of the women) were perfume, nail polish and eye pencil. There was no difference between the subgroups in the proportions of women who cited ingredients as the criterion of choice (results not shown).

### 3.4. Proportion of Women Intending to Change (NPW) and Having Changed (PW) Cosmetics Use during Pregnancy

**Figure 2:**
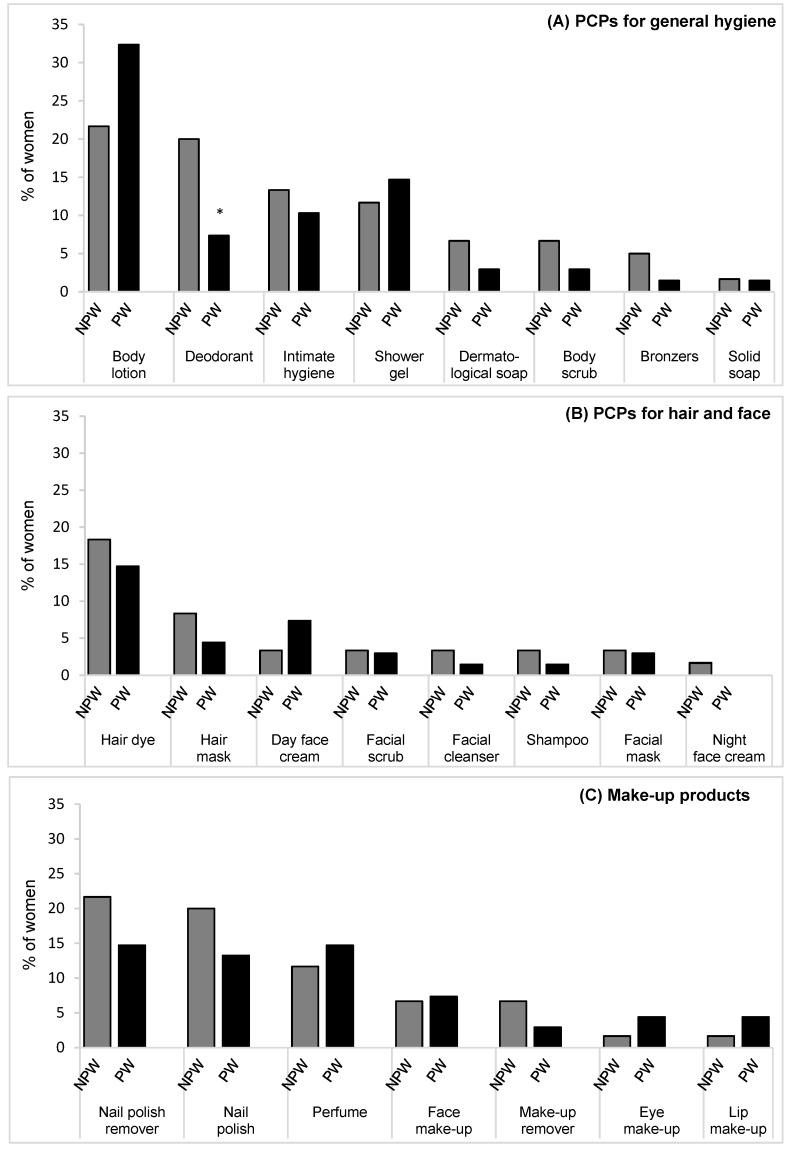
Proportion of women intending to change (NPW) or having changed (PW) the use of cosmetics during pregnancy. (**A**) PCPs for general hygiene; (**B**) PCPs for hair and face; (**C**) make-up products. * *p* = 0.04. Abbreviations: Eye make-up, eyeliner, eye shadow, eye pencil and mascara; Face make-up, foundation make-up and blush; Lip make-up, lip pencil and lipstick; NPW: non-pregnant women; PW: pregnant women.

The intention of changing deodorant use was significantly more common among the NPW than among the PW subgroup (20.3% *vs.* 7.4%) (*p* = 0.04). Changes or intended changes in the use of other PCPs and make-up products during pregnancy did not differ between the two subgroups. The most frequent changes in use were of body lotion, nail polish, nail polish remover and hair dye (Figure 2).

### 3.5. Types of Changes Made during Pregnancy

**Figure 3:**
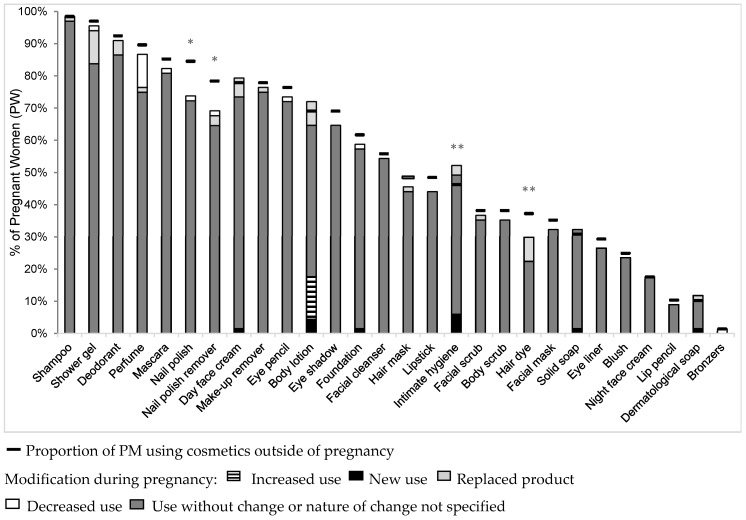
Proportion of pregnant women (PW, *n* = 68) using cosmetics outside and during pregnancy. * *p* < 0.05; ** *p* ≤ 0.1; Abbreviations: PW: pregnant women.

Changes in cosmetics use in the PW subgroup during pregnancy are shown in Figure 3. There were significantly fewer women in this subgroup using nail polish (*p* = 0.02) and nail polish remover (*p* = 0.04) during pregnancy compared with the proportions of users before pregnancy. The products most commonly given up by PW were nail polish (10.3%), nail polish remover (7.4%) and hair dye (7.4%) while perfume was the one whose use was the most often decreased (10.3%). Cosmetics most often replaced by others considered to be less harmful were shower gel (10.3%), body lotion (7.4%) and hair dye (7.4%). Some women used products that they had not previously used: body lotion, intimate hygiene product (4.4% each), day face cream, dermatological soap, and solid soap and make-up foundation (1.5% each). Body lotion was the only cosmetic whose use increased (13.4%) (Figure 3).

For other cosmetics commonly used during pregnancy, the proportions of NPW intending to use massage oil and gel for tired aching legs in the event of pregnancy (54.4% and 57.1%, respectively) were significantly higher than those of the PW actually using the products (26.2% and 11.3%, respectively) (*p* < 0.001). There was no difference between the two subgroups in the use or intended use of striae gravidarum cream (75.2%).

### 3.6. Criteria of Choice of Cosmetics during Pregancy

**Figure 4:**
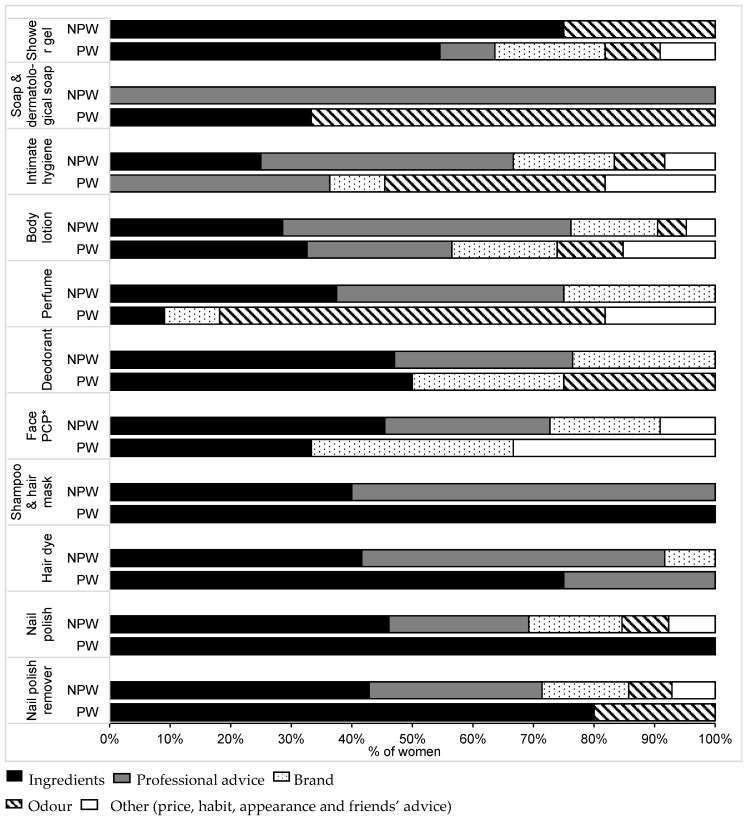
Criteria of choice of cosmetics during pregnancy. The percentages were calculated only in the subgroups of NPW who declared an intention to change cosmetics use during pregnancy. Shower gel (*n* = 7), solid soap and dermatological soap (*n* = 4), intimate hygiene product (*n* = 8), body lotion (*n* = 13), perfume (*n* = 7), deodorant (*n* = 12), face PCP (*n* = 9), shampoo and hair mask (*n* = 6), hair dye (*n* = 11), nail polish (*n* = 12) and nail polish remover (*n* = 13). The percentages were calculated only in the subgroup of PW having changed cosmetics use during pregnancy. Shower gel (*n* = 10), solid soap and dermatological soap (*n* = 3), intimate hygiene product (*n* = 7), body lotion (*n* = 22), perfume (*n* = 10), deodorant (*n* = 5), face PCP (*n* = 9), shampoo and hair mask (*n* = 3), hair dye (*n* = 10), nail polish (*n* = 9) and nail polish remover (*n* = 10). * Face PCP corresponds to facial cleanser, day face cream, night face cream, facial scrub, make-up remover and foundation. Abbreviations: NPW: non-pregnant women, PW: pregnant women.

Few in the PW subgroup changed their cosmetics use, and, if so, the new criteria of choice were mainly safe product ingredients and odor. In the event of pregnancy, the NPW stated that any intended change would be governed by ingredients and professional advice (Figure 4).

### 3.7. Risk Perception and Advice from Health Professionals

**Figure 5:**
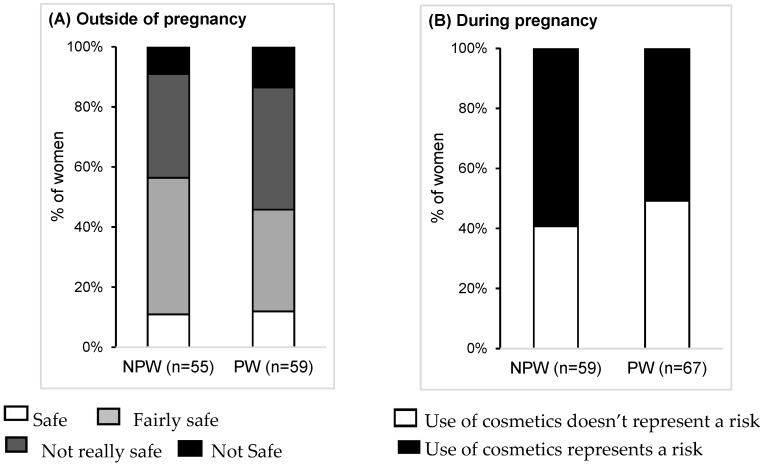
Risk perception related to cosmetic products. (**A**) Outside of pregnancy; (**B**) During pregnancy. All *p*-values >0.05. Abbreviations: NPW: non-pregnant women; PW: pregnant women.

The women in the study had contrasting perceptions of risk related to cosmetics. Outside of pregnancy, cosmetics were generally seen as “fairly safe” (39.5%) or “not really safe” (37.7%). More than half of the women (54.8%), with no significant difference between the subgroups, thought that there was a risk in using cosmetics during pregnancy (Figure 5).

**Figure 6:**
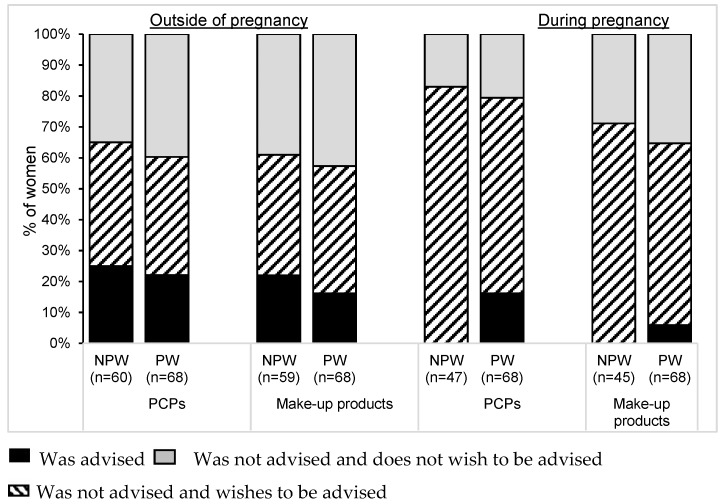
Advice received from health professionals about cosmetics. Outside of pregnancy: proportion of women having received advice (NPW and PW). During pregnancy: proportion of women wishing to receive advice (NPW) or having received advice (PW). All *p*-values > 0.05. Abbreviations: NPW: non-pregnant women; PCPs: personal care products; PW: pregnant women.

Figure 6 shows the proportion of women who had received advice from a health professional about the use of cosmetics outside of and during pregnancy. Outside of pregnancy, only a minority of women had received advice about PCPs (23.4%) or make-up products (18.9%). During pregnancy, 16.2% of the PW had received advice about PCPs and 5.9% about make-up products. Among the women who had not received advice, half would have liked some guidance outside of pregnancy (51.0% about PCPs and 49.5% about make-up products), and greater numbers would have appreciated advice during pregnancy itself (78.8% about PCPs and 66.1% about make-up products) (Figure 6).

## 4. Discussion

### 4.1. Use of Cosmetics Outside of Pregnancy

The cosmetics use habits outside of pregnancy of the women in our study are overall similar to those reported in other studies of adult women in Europe [34,35] and the USA [39]. However, the women in our study declared a greater use of make-up products (foundation, eye make-up, nail polish and nail polish remover) than in the Dutch study of Biesterbos *et al.* [34] and greater use of perfume and shower gel than in the American study of Wu *et al.* [39]. In contrast with women in other studies, however, they used less body lotion [35,39], night facial cream [34], and hair dye [34,39].

### 4.2. Use of Cosmetics during Pregnancy

In our study, the cosmetics use habits of the PW were comparable to those of pregnant women in other studies performed in the USA and in Canada for body lotion [16,36,37], deodorant [16,36], make-up foundation [16,17], facial cleanser [17,37] and lipstick [16]. In contrast, more pregnant women in our study, compared to those in these other studies, used perfume, shampoo, shower gel, face creams, hair dye, eye make-up products, nail polish and nail polish remover [11,16,17,36,37]. The data collected in the other studies concerned cosmetics use during the 24 h [11,16] or 48 h [17,36] before the participants replied to the survey questionnaire, whereas our data represented overall use habits. The proportions of women using cosmetics in the studies carried out in the USA and in Canada may have been underestimated, in particular with regard to products not used daily such as hair products, nail polish and perfume. In addition, the life habits of American women differ from those of their counterparts in France and the rest of Europe. Caution should therefore be taken when making direct comparisons of the different findings. The proportion of women in our study using preventive treatment of striae gravidarum (75%) was comparable to that of another French study [40].

### 4.3. Perception of Risk Related to Cosmetics and Their Ingredients

Almost half of the women (45%) in our study considered that there was no risk in using cosmetics during pregnancy. However, it is now recognized that these products contain numerous potentially harmful chemical substances including plasticizers, bisphenol A, parabens, synthetic dyes, benzophenones, antimicrobials, dioxane, formaldehyde and heavy metals [1,2,3,4,5,6]. Some of these molecules, such as formaldehyde and dioxane, are known carcinogens or probably carcinogenic [41,42], and certain synthetic dyes are suspected of being carcinogenic [43]. In Europe, a red azo dye (colour index number 18050), suspected of being carcinogenic and genotoxic, has been banned for use in food [44] but not in cosmetics [1]. Other molecules are endocrine disruptors in humans. Exposure in utero to phthalates and phenols has been related to impaired male genital development [28,29,45]. Exposure to TPHP, a plasticizer found particularly in nail polish [18], has been related to a decrease in sperm concentration [30]. Cosmetics users are largely unaware of these findings. Our study shows that outside of pregnancy, product ingredients, except for a few PCPs (dermatological soap and night facial cream, shower gel and deodorant), were not our participants’ main criterion of choice of cosmetics. During pregnancy, in contrast, changes in use were in most cases dictated by the ingredients of the cosmetics. However, the value of these conclusions is limited by the small number of women who made changes to their use habits.

### 4.4. Impact of Pregnancy on the Risk Perception of Cosmetics

Our study participants changed their use habits during pregnancy for only five out of 28 cosmetics (hair dye, deodorant, nail polish, nail polish remover and perfume). In the PW subgroup, the changes consisted mainly in replacing certain PCPs with other less harmful products and in stopping or reducing the use of make-up products. Only two products, nail polish and nail polish remover, were used significantly less by the PW during pregnancy than before. A review of the literature showed that no other study has investigated the change in cosmetics use during pregnancy (compared to the period before pregnancy). Our study shows the impact of pregnancy on the perception of risk related to cosmetics. In a recent study, Lang *et al.* (2016) [37] found that use of cosmetic and hair styling products decreased as pregnancy progressed, whereas that of general hygiene and skincare products was consistent across time periods. However, they had no information on the prevalence of use of these products before pregnancy.

Our study is novel in its comparison of cosmetic use and the perception of risk of cosmetic products in two subgroups of women (PW and NPW). Another study concurrently performed on the same subgroups of PW and NPW showed that the former had a more considered approach to self-medication [46]. They did not show a similar caution, however, when it came to cosmetics since subjects in the NPW subgroup would be more inclined to change their deodorant use in the event of pregnancy and also stated that they intended to use massage oils and gel for tired aching legs more frequently. These discrepancies in behavior could be due to a lack of awareness about these PCPs. Indeed, our study shows that informing and advising women about products used during pregnancy would have an overall beneficial effect on health.

### 4.5. Need of Advice from Health Professionals about the Safe Use of Cosmetics

Few women taking part in the study had been given advice by a health professional about the use of cosmetics either before or during pregnancy (16% for PCPs and 6% for make-up products). However, more than 65% of women would have liked to receive information, in particular during pregnancy. These results reflect the desire and need of women to be advised about their exposure when pregnant to the chemical substances contained in cosmetics. All perinatal health professionals should be aware of the potential risks of the use of these products during pregnancy and be ready to give appropriate advice. A recent study on household chemicals reported that pregnant women had greater confidence in information given to them by a physician than in articles on Internet or the opinions of friends [47]. Cosmetics are increasingly sold in pharmacies, and community pharmacists are therefore in a unique position to inform and advise pregnant clients. In France, moreover, their educational role in health and prevention is acknowledged by law, a role recently strengthened by the Hospital, Patients, Health and Territories (HPST) Law [48,49].

In some European countries (in particular France and Denmark), the health authorities inform women about exposure to chemicals resulting from the use of cosmetics during pregnancy [50,51]. To avoid unnecessary and potentially dangerous use of cosmetics women should be encouraged to use them less often and to decrease the amounts applied. Certain beauty products such as nail polish, nail polish remover and hair dye could be eliminated altogether during pregnancy. Others could be replaced by products that contain fewer chemical substances or are less readily absorbed. For example, Delmaar *et al.* observed that use of spray deodorants led to greater exposure to phthalates than that of non-spray deodorants [52]. The accumulation of absorption routes (inhalation and cutaneous absorption in the use of spray deodorants) could also increase the exposure levels of some other substances and not specifically phthalates. Further studies are needed to confirm this hypothesis. Finally, creams purporting to limit the occurrence of striae gravidarum have not had their efficacy and safety tested and proven. These products should not be recommended for all women, therefore, but reserved for those with risk factors such as a family history of striae, prepregnancy overweight and increased weight gain during pregnancy [40,53].

### 4.6. Limitations

Our study has several limitations. It carries a risk of selection bias because the participants were volunteers and may therefore have been more alert to the issues involved. No record was made of the number of women who declined to take part. The women in our study, compared to those in a French perinatal survey in 2010 [54] or in a French perinatal database in 2011 [55], had a higher level of education (university level: 75% *vs.* 52%) and more jobs in executive and intellectual professions (22% *vs.* 16.5%) or intermediate professions (36% *vs.* 28%). However, the proportion of women in France with higher education qualifications and positions in intellectual and intermediate professions had increased over time [54,55]. It is likely that this trend continued up to 2015 (the year of our study), which would thus minimize the differences observed. Level of education and income had an influence on the prevalence of use of many PCPs [34,35,37,39]. For example, the use of body lotion, hand cream, hair spray and deodorant decreased with increasing levels of education, whereas that of make-up products, facial cleansers and sunscreens increased [34,35,39]. The perception of risk of environmental chemicals by pregnant women is also related to educational level [38]. Thus, our results are to be treated with caution and further studies more representative of the general population are needed before any general conclusions can be drawn. Social desirability bias may have affected the women’s self-reported use of PCPs, in particular for the questions about changes in use during pregnancy. Although this bias is more frequent for sensitive areas such as illicit drug use and sexual behavior [56], it is possible that our participants were more likely to declare that they had changed or decreased their use of PCPs as evidence of responsible behavior and proper care of their unborn child. This bias seemed to be limited in our study, however, since few women declared they had changed cosmetics use during pregnancy. Conversely, the possibility of under-reporting of increased use of products cannot be excluded. In addition, our cross-sectional design did not assess the use of drugs and other products throughout pregnancy. There is therefore potentially a risk of recall bias concerning these habits, particularly for pregnant women at a gestational age close to term. This bias could lead to an under-estimation of the use of some products. It is also possible that at the beginning of pregnancy women do not consume the same products as women in their second or third trimester (e.g., cream for striae gravidarum, gel for tired aching legs). However, 90% of the PW subgroup were in their second or third trimester of pregnancy, which could limit the differences in use.

## 5. Conclusions

To our knowledge, this study is the first to investigate changes in cosmetics use during pregnancy. The results showed that few women stopped use during pregnancy, with the exception of nail polish and nail polish remover. Almost half of the participants considered the use of cosmetics without risk during pregnancy, and few had received advice on the matter by a health professional. Studies involving samples more representative of the general population of pregnant women are needed to confirm our findings. Nevertheless, our observations would be useful in making perinatal health professionals more aware of the problems raised by cosmetics use during pregnancy. All health professionals should be ready to inform and advise women about the potential risks and the safety of use of these products.

## Figures and Tables

**Table 1:** Socio-demographic and obstetric characteristics of women (*n* = 128).

Personal Information	Total *N* = 128 (%) (m ± SD) ^a^	NPW Group *N* = 60 (%) (m ± SD) ^a^	PW Group *N* = 68 (%) (m ± SD) ^a^	*p*-Value
Age	*n* = 127	*n* = 60	*n* = 67	-
in years	30.5 ± 5.8	30.7 ± 7.4	30.2 ± 3.9	0.67
Town of residence	*n* = 124	*n* = 57	*n* = 67	-
≤5000 inhabitants	82 (66.1)	30 (52.6)	52 (77.6)	<0.01
>5000 inhabitants	42 (33.9)	27 (47.4)	15 (22.4)	-
Educational level	*n* = 127	*n* = 59	*n* = 68	-
Junior school	8 (6.3)	5 (8.5)	3 (4.4)	0.70
High school	24 (18.9)	11 (18.6)	13 (19.1)	-
University level	95 (74.8)	43 (72.9)	52 (76.5)	-
**Socioprofessional category** ^b^	*n* = 128	*n* = 60	*n* = 68	-
Executive and intellectual professions	28 (21.9)	13 (21.7)	15 (22.1)	1.00
Intermediate professions ^c^	46 (35.9)	21 (35.0)	25 (36.8)	-
Employees	40 (31.3)	19 (31.7)	21 (30.9)	-
Others ^d^	4 (3.1)	2 (3.3)	2 (2.9)	-
No occupation	10 (7.8)	5 (8.3)	5 (7.4)	-
**Previous pregnancy**	*n* = 127	*n* = 60	*n* = 67	-
≥1	65 (51.2)	32 (53.3)	33 (49.3)	0.78
**Chronic treatment** ^e^	*n* = 128	*n* = 60	*n* = 68	-
Outside of pregnancy	19 (14.8)	13 (21.7)	6 (8.8)	0.05

^a^ m ± SD: mean ± standard deviation; ^b^ Socioprofessional categories are presented according to the French nomenclature (Institut national de la statistique et des études économiques); ^c^ Intermediate professions comprises education (e.g., teachers), health (e.g., nurses) and public services; ^d^ Others: Artisans, retailers and company head (*n* = 1), manual workers (*n* = 2), student (*n* = 1); ^e^ Chronic treatments for NPW group: Allopathic treatment (ebastine (*n* = 2), rosuvastatin (*n* = 1), venlafaxine (*n* = 1), escitalopram (*n* = 1), proton-pump inhibitor (*n* = 1), interferon beta-1a (*n* = 1), chlormadinone and topiramate (*n* = 1), modafinil and sodium oxybate (*n* = 1), zinc gluconate, benzoyl peroxid and adapalene (*n* = 1), rivaroxaban, thiamazole and levothyroxine (*n* = 1)), homeopathic treatment (*n* = 2); Chronic treatments for PW group: zolmitriptan (*n* = 1), duloxetine and valproic acid (*n* = 1), ebastine and budesonide (*n* = 1), levothyroxine (*n* = 1), levothyroxine and cetirizine (*n* = 1), dietary supplement (*n* = 1). NPW: non-pregnant women; PW: pregnant women.

**Table 2:** Proportion of women reporting regular use of personal care products outside of pregnancy (*n* = 128).

Personal Care Product	Total *n* = 128 (%)	NPW Group *n* = 60 (%)	PW Group *n* = 68 (%)	*p*-Value
**General hygiene**				
Shower gel	120 (93.7)	54 (90.0)	66 (97.1)	0.15
Solid soap	45 (32.5)	24 (40.0)	21 (30.9)	0.37
Dermatological soap ^a^	22 (17.5)	15 (25.9)	7 (10.3)	0.03
Intimate hygiene product ^a^	58 (46.0)	27 (45.8)	31 (46.3)	1
Body scrub	51 (39.8)	25 (41.7)	26 (38.2)	0.83
Body lotion	92 (71.9)	46 (76.7)	47 (69.1)	0.45
Deodorant ^b^	117 (92.1)	55 (91.7)	62 (92.5)	1
Perfume ^b^	116 (91.3)	56 (93.3)	61 (91.0)	0.75
Bronzers	1 (0.8)	0	1 (1.5)	
**Face hygiene**				
Facial cleanser ^a^	77 (60.6)	39 (66.1)	38 (55.9)	0.32
Day face cream	105 (82.0)	52 (86.7)	53 (77.9)	0.25
Night face cream	24 (18.7)	12 (20.0)	12 (17.7)	0.91
Facial scrub	56 (43.7)	30 (50.0)	26 (38.2)	0.25
Facial mask	45 (35.2)	21 (35.0)	24 (35.3)	1
**Hair hygiene**				
Shampoo	126 (98.4)	59 (98.3)	67 (98.5)	1
Hair mask	55 (43.0)	22 (36.7)	33 (48.5)	0.24
Hair dye ^a^	43 (34.1)	18 (30.5)	25 (37.3)	0.55

^a^ 2 missing responses; ^b^ 1 missing response. NPW: non-pregnant women; PW: pregnant women.

**Table 3:** Proportion of women reporting regular use of make-up products outside of pregnancy (*n* = 128).

Make-Up Product	Total *n* = 128 (%)	NPW Group *n* = 60 (%)	PW Group *n* = 68 (%)	*p*-Value
**Make-up foundation**	74 (57.8)	32 (53.3)	42 (61.8)	0.43
**Blush**	36 (28.1)	19 (31.7)	17 (25.0)	0.52
**Mascara**	110 (85.9)	52 (86.7)	58 (85.3)	1
**Eye-liner**	43 (33.6)	23 (38.3)	20 (29.4)	0.35
**Eye pencil**	93 (72.7)	41 (68.3)	52 (76.5)	0.41
**Eye shadow**	88 (68.7)	41 (68.3)	47 (69.1)	1
**Lipstick**	58 (45.3)	25 (41.7)	33 (48.5)	0.55
**Lip pencil** ^a^	12 (9.4)	6 (10.2)	7 (10.5)	1
**Make-up remover**	104 (81.3)	51 (85.0)	53 (77.9)	0.38
**Nail polish**	96 (75.0)	41 (68.3)	55 (80.9)	0.15
**Nail polish remover** ^b^	92 (73.6)	41 (68.3)	51 (78.5)	0.28

**^a^** 1 missing response; **^b^** 3 missing responses; NPW: non-pregnant women; PW: pregnant women.

## Abbreviations

The following abbreviations are used in this manuscript:

EU	European Union
NPW	non-pregnant women
PCP	personal care products
PW	pregnant women
TPHP	Triphenyl phosphate
